# Vulnerability assessment model integrating outcome and characteristic-based metrics for electric motorcycle battery swapping and charging stations

**DOI:** 10.1038/s41598-025-20325-x

**Published:** 2025-10-21

**Authors:** Yusuf Priyandari, Wahyudi Sutopo, Muhammad Nizam, Hendro Wicaksono

**Affiliations:** 1 Industrial Engineering Department, Faculty of Engineering, Universitas Sebelas Maret, Surakarta, 57126 Indonesia; 2 Electrical Engineering Department, Faculty of Engineering, Universitas Sebelas Maret, Surakarta, 57126 Indonesia; 3 School of Business, Social and Decision Sciences, Constructor University, Campus Ring 1, Bremen, 28759 Germany

**Keywords:** Battery swapping and charging station, Electric motorcycle, Operation transaction data, Product service system, Vulnerability assessment, Engineering, Information technology

## Abstract

Battery swapping and charging stations are essential for increasing the adoption of electric motorcycles. The stations address the range anxiety issue and quickly obtain a fully recharged battery. However, operational issues with swapping and charging activities drive operational vulnerability. Therefore, this study proposes a vulnerability assessment model utilizing the IoT Platform data of electric motorcycle battery swapping and charging stations. The model computes a vulnerability score by integrating vulnerability indicator metrics of the system outcome and characteristic. The system outcome uses performance data representing vulnerability impact. The system characteristic uses data from the vulnerability driver and exposure factors. The driver factor represents mitigation ability, and the exposure factor represents conditions that may affect both the mitigation ability and performance. The model also classifies the vulnerability of stations in four categories: not vulnerable, potentially vulnerable, moderately vulnerable, and vulnerable. The model was implemented in a case in Jakarta. The result reveals significant differences in vulnerability among stations, although most stations fall into the not vulnerable to moderately vulnerable categories. The findings facilitate identifying station characteristics that potentially affect performance quantitatively.

## Introduction

Battery swapping and charging stations are crucial infrastructure for increasing the adoption of electric vehicles^1–4^. They address the issues of range anxiety and quickly obtain a fully recharged battery^2,5^. The infrastructure has also spurred innovations in plug-in electric vehicle development, enabling vehicle-to-grid energy return^6,7^, mobile swapping and charging stations^8^, and other innovative charging and swapping stations^9^. Indonesian regulations differentiate between charging stations and battery swapping stations for electric vehicles^10–12^. In practice, charging stations are typically built for electric cars, while swapping stations are designed for electric motorcycles. However, battery swapping stations for electric motorcycles are now used as charging stations to improve station utilization and enhance customer services.

**Fig. 1:**
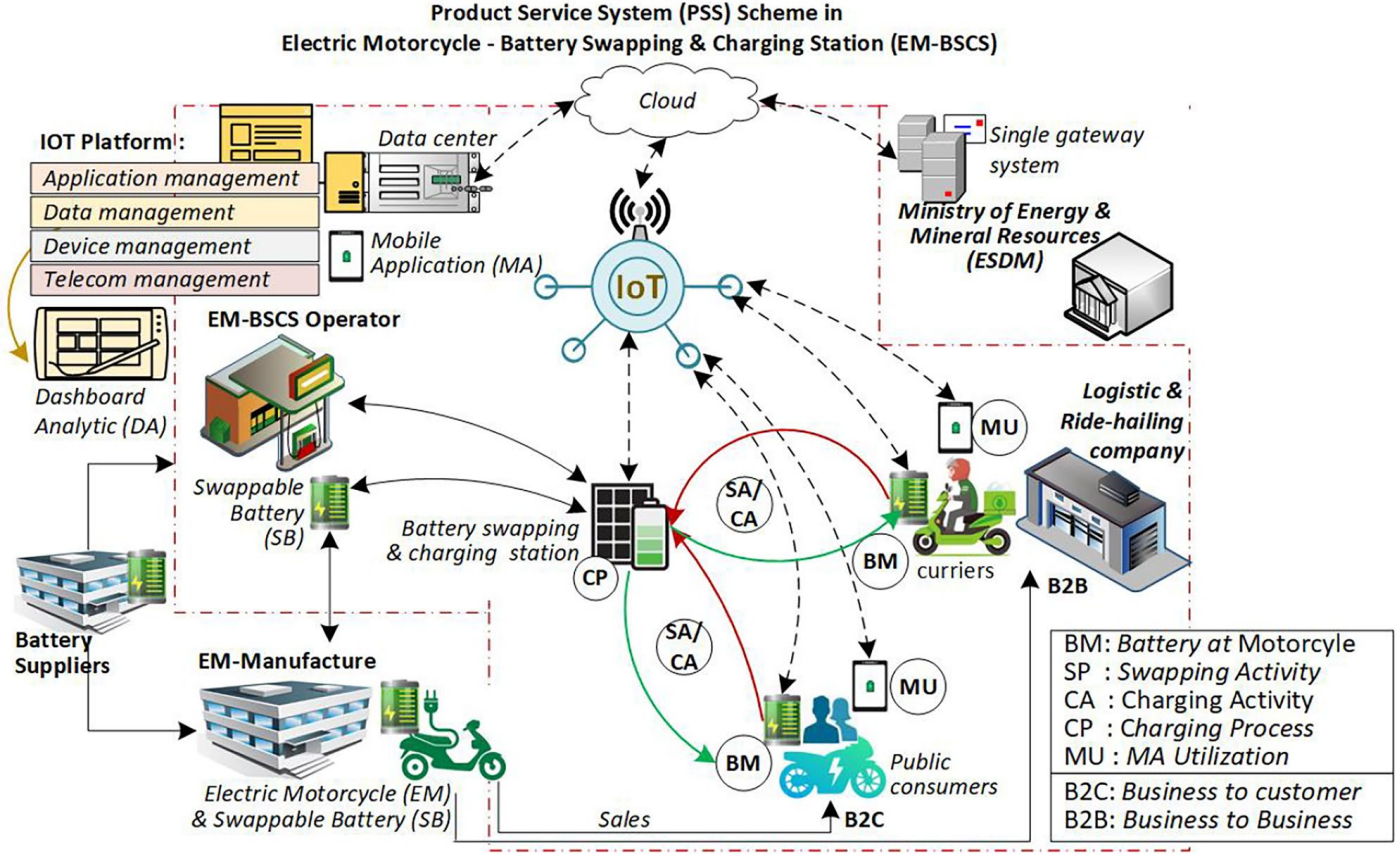
Product service system (PSS) in the EM-BSCS.

Activities at the electric motorcycle battery swapping and charging station (EM-BSCS) are illustrated in Fig. 1. Electric motorcycle owners receive a battery from the manufacturer upon purchasing the vehicle or may lease a battery from the company operating the EM-BSCS. The company is responsible for inventory planning, battery allocation across all stations, and site planning. Each station operates its battery swapping and charging services autonomously. Consumers use a mobile application to carry out battery swapping or charging transactions at the station. In a battery swapping system, battery ownership is considered public, as batteries are transferred from one user to another. In contrast, battery ownership remains private during a charging system and is retained by the vehicle owner.

From a supply chain perspective, the EM-BSCS forms a closed-loop, service-centric digital supply chain that involves asset circulation (batteries), customer demand, infrastructure utilization, and reverse logistics (battery return and charging)^13,14^. Key operational elements such as inventory balancing, station throughput, charging delays, and service disruptions are tightly interconnected, similar to conventional supply chain systems. Thus, disruptions or inefficiencies at any node (station) can propagate throughout the network^15^. The links between the charging station operator, charging equipment, and electric vehicle are the most vulnerable in the electric vehicle ecosystem^16^.

A preliminary data analysis from the EM-BSCS company revealed 1,595 operational issues in 225 stations from October to December 2023. These issues (e.g., delays due to server issues, damaged socket, unrecognized battery ID, and offline station) may impair the performance of battery swapping services. Furthermore, user behavior such as swapping at a critically low state of charge (SOC) may contribute to battery degradation, extended charging durations, and further compromise performance issues. The analysis also reveals variation in daily swapping throughput, with occasional sharp declines at specific periods. These conditions indicate potential operational vulnerabilities within the EM-BSCS system.

Failure to assess and monitor the vulnerability of EM-BSCS may result in undetected performance degradation (e.g., prolonged service downtime or low throughput), inefficient resource allocation (e.g., batteries or cabinets), and weakened customer trust. The performance degradation could harm the system’s ability to serve consumers effectively^16,17^. Such outcomes can undermine the reliability and scalability of EM-BSCS infrastructure, ultimately threatening electric motorcycle adoption and sustainable urban mobility goals.

Given these circumstances, vulnerability assessment is essential in the context of the EM-BSCS, as the system is exposed to potential risks including natural disruptions, cybersecurity threats, and technical failures^18–24^. The operational vulnerabilities of the EM-BSCS system have not been examined in prior research. Most studies investigate vulnerabilities in electric vehicle (car) charging systems^16,18–21,23,25^ and the management battery system^24^. The identified issues relate to cyber-physical security^16,19,21,23,25^, service failures and inconveniences^20^, and the vulnerability of electric road networks vulnerability^18^. There is also a study about supply chain vulnerability affected by electric vehicle battery chemistry^26^. Therefore, this study addresses the research gap on vulnerabilities for EM-BSCS by focusing on operational vulnerability aspects, not cyber-physical security issues.

Vulnerability studies often encounter limitations due to the unavailability of quantitative data^16^. Encouragingly, the EM-BSCS service system, as depicted in Fig. 1, also constitutes an example of a Product-Service System (PSS)^27–30^. The electric motorcycle manufacturer extends beyond product sales by developing battery swapping and charging services through the EM-BSCS, which is managed by a dedicated company. This company provides a mobile application for consumers, deploys IoT-enabled stations, and manages service data via an IoT platform. The IoT platform^29,31–33^, which handles big data, facilitates service monitoring, and supports decision-making for the company through big data analytics^2,34–38^ or supply chain analytics^13^. As the EM-BSCS represents a Product-Service System (PSS) model enriched with big data from the IoT Platform, this study utilizes such data to assess system vulnerability quantitatively.

Therefore, this study proposes a comprehensive vulnerability assessment model utilizing the IoT Platform data and focusing on EM-BSCS operation, not cyber-physical security issues. The model innovation lies in calculating a vulnerability score by integrating vulnerability indicator metrics of the system outcome and characteristic. The concept of vulnerability proposed will be outlined in the literature review, and the vulnerability indicator metrics of the system outcome and characteristic will be outlined in the method. The vulnerability score is calculated by extracting data from swapping and charging transactions, station profiles, and inherently relevant external data. Indicators or attributes proposed for calculating the vulnerability score will be outlined in the subsection “Performance vulnerability indicator, Vulnerability Driver factor, and Vulnerability exposure factor”.

To the best of our knowledge, our work is the first quantitative vulnerability assessment model specifically developed for EM-BSCS. Unlike existing studies focusing on electric vehicle (car) infrastructure or cyber-physical vulnerabilities, our model focuses on operational vulnerability and uniquely integrates outcome-based metrics with characteristic-based metrics using real-world IoT data from EM-BSCS operations. This dual-layer approach enables more granular, site-specific resilience evaluation, offering a novel contribution to operational monitoring and decision support in the EM-BSCS domain. The concept of vulnerability, metrics for calculating a score of vulnerability, and the vulnerability indicators will be outlined in the literature review and the method.

The rest of this paper is organized as follows. As documented in prior research, the “Literature” section provides a comprehensive overview of existing definitions, models, and methods for measuring vulnerability. It also includes It also includes the specific definitions and the model proposed. The “Methodology” section outlines the vulnerability performance metric, the indicators definition and their measurement unit employed, the data normalization method and the formulas used to compute the vulnerability score. The “Results” section presents insights from a case study on estimating the vulnerability scores for EM-BSCSs. Finally, the “Discussion” section evaluates the strengths and limitations of the assessment model and summarizes the conclusions drawn from the research findings.

## Literature review

We reviewed several studies that explore the vulnerabilities of battery swapping and charging systems for electric motorcycles. However, the majority of existing research focuses on vulnerabilities in electric vehicle (EV) charging systems^16,18–21,23,25^ and battery management systems^24^. Furthermore, the studies predominantly focus on topics such as cyber-physical security^16,19,21,23,25^, service disruptions^25^, and electric road networks vulnerability^18^. For example, Glombek^20^ investigated vulnerabilities in electric vehicle charging stations, identifying service failure and inconvenience vulnerabilities to determine critical sites and support decisions on optimal station placement. Reeh^19^ studied vulnerabilities in electric vehicle charging infrastructure by defining several potential failure scenarios and analyzing the impacts of possible cyber-physical attacks. Meanwhile, Ke Zhang^18^ examined vulnerabilities in Electric Road Networks (ERNs) concerning attack intentions and methods for identifying critical fast charging stations within the network.

In contrast to those studies, this research aims to develop a vulnerability assessment model focused on the operational aspects of the EM-BSCS, leveraging data from the IoT Platform and thus necessitating a broader conceptualization of vulnerability. Therefore, we review vulnerability concepts within supply chain systems. This aligns with the introduction, positioning the EM-BSCS as a closed-loop and centric digital supply chain model.

### A vulnerability concept in cyber-physical security

NIST^39^ defines a vulnerability as a flaw or weakness within an information system, security procedures, internal controls, or implementation that can be exploited by threats, potentially resulting in high risks. For example, human threats to cyber-physical security and insufficient procedures to address such threats constitute vulnerabilities. The vulnerability may also be driven by a predisposing condition that exists within an organization (e.g., organizational governance structures, dependencies on supply chains, information technologies, and telecommunications providers, and enterprise security architectures).

The definition highlights that vulnerability encompasses inherent system weaknesses susceptible to threat exploitation, elevating risk levels, and predisposing organizational conditions that act as drivers of vulnerability. Reeh^19^ use the concept from NIST^39^ for vulnerability analysis and risk assessment in electric vehicle charging infrastructure, focusing on cyber-physical threats.

### Broader concept of vulnerability

Hashim^40^ defines supply chain vulnerability as the potential for loss within the supply chain. FAO^41^ characterizes vulnerability as a condition under which the system is at risk. Elleuch et al.^42^ stated that vulnerability is a condition in which exogenous factors (e.g., disturbances and disruptions) easily influence a risk’s intensity. Wagner and Bode^43^ and Wagner and Neshat^44^ defined vulnerability as a function of supply chain characteristics and disruptions that dictate the extent of losses. These definitions view vulnerability as being confined to risks or loss.

Sharma et al.^45^ emphasize vulnerability as a system complexity includes supply chain (e.g., number of nodes and node criticality), organization (e.g., product and process), supply chain relationship (e.g., type and degree of alignment), and information management (e.g., information visibility and warning system) that can disrupt a manufacturing system. Deshpande^46^ describes vulnerability as a latent characteristic in the supply chain that becomes apparent through the increased frequency and severity of disruptions in the supply chain. Both definitions constitute internal conditions as vulnerability drivers.

Moret and ASPIRES^47^ distinguished between risk and vulnerability by stating that risk is a condition of several vulnerability indicators that may cause a risk and lack the ability of the system to make a corrective response. Nowakoski et al.^48^ define vulnerability as a condition wherein system performance is prone to decline due to limited coping capacity to threats. These definitions emphasize a system’s inability to mitigate threats, which can lead to decreased performance. For example, the inability of the production department to anticipate the breakdown of some machines results in order delays. External disruptions, such as floods, cause delays in receiving raw materials, potentially leading to additional impact on fulfilling orders.

Other researchers such as Vlajic^49^ focuse on situations where the performance value falls outside the expected (robustness) range as a vulnerability condition. Despande^46^ extends Vlajic’s concept^49^ as a state in which performance remains outside the robustness range, neither improving nor stabilizing, but instead continuing to deteriorate. The definition highlights that vulnerability occurs when performance deviates beyond the expected range and does not recover, but instead progressively declines.

### Vulnerability assessment

In an information security context, vulnerability assessment is an activity to understand the nature and degree to which organizations, business processes, and information systems are vulnerable to threat sources^39^. In cyber-physical systems, the degree of vulnerabilities is often assessed based on the number of potential threats and the magnitude of the impact resulting from their exploitation, using methods such as STRIDE^25^ or the Common Vulnerability Scoring System (CVSS)^16,50^.

Insufficient information on possible system threats often necessitates conducting vulnerability assessments in a qualitative or semi-quantitative manner^16^. For example, vulnerability is often measured through indices of the risk severity, occurrence (frequency of events), and detectability (ease of detection). The measurement model, such as Failure Mode and Effect Analysis (FMEA), Cause-Consequence Analysis (CCA), Supply Chain Event Management (SCEM), and Supply Chain Risk Management (SCRM)^48^. Some assessment is manifested as resilience to the system; therefore, the assessment evaluates readiness (how prepared a system is to handle vulnerable conditions), responsiveness (how quickly a system reacts to vulnerable conditions), and recovery (the system’s ability to return to normal after disruptions)^48^. To determine the readiness value, researchers such as Nowakowski^48^ develop a vulnerability index from qualitative opinions of stakeholders.

In contrast to Nowakowski^48^, Vlajic^49^ introduced a quantitative assessment model, namely VULA. The model utilizes the deviation of performance from an expected value and counts the frequency of the deviations. In line with the VULA, Key Risk Indicator (KRI) also uses relevant metrics that provide insight into potential risks that may impact system performance^51^ and the Supply Chain Vulnerability Index (SCVI) that uses graph theory to calculate the vulnerability index^52^. In the electric vehicle charging infrastructure, Glombek^20^ developed a vulnerability measurement framework encompassing service failure vulnerability and inconvenience vulnerability. Those studies focus on measuring vulnerability through the vulnerability impact or outcome-based approach.

Therefore, to leverage data availability from the IoT Platform, this study develops a quantitative vulnerability assessment model that computes scores using vulnerability indicator metrics encompassing system outcomes and characteristics. This model is based on the vulnerability concept previously described and illustrated in Fig. 3. To our knowledge, few studies have integrated outcome and characteristic-based approaches to vulnerability measurement.

## Methodology

**Fig. 2:**
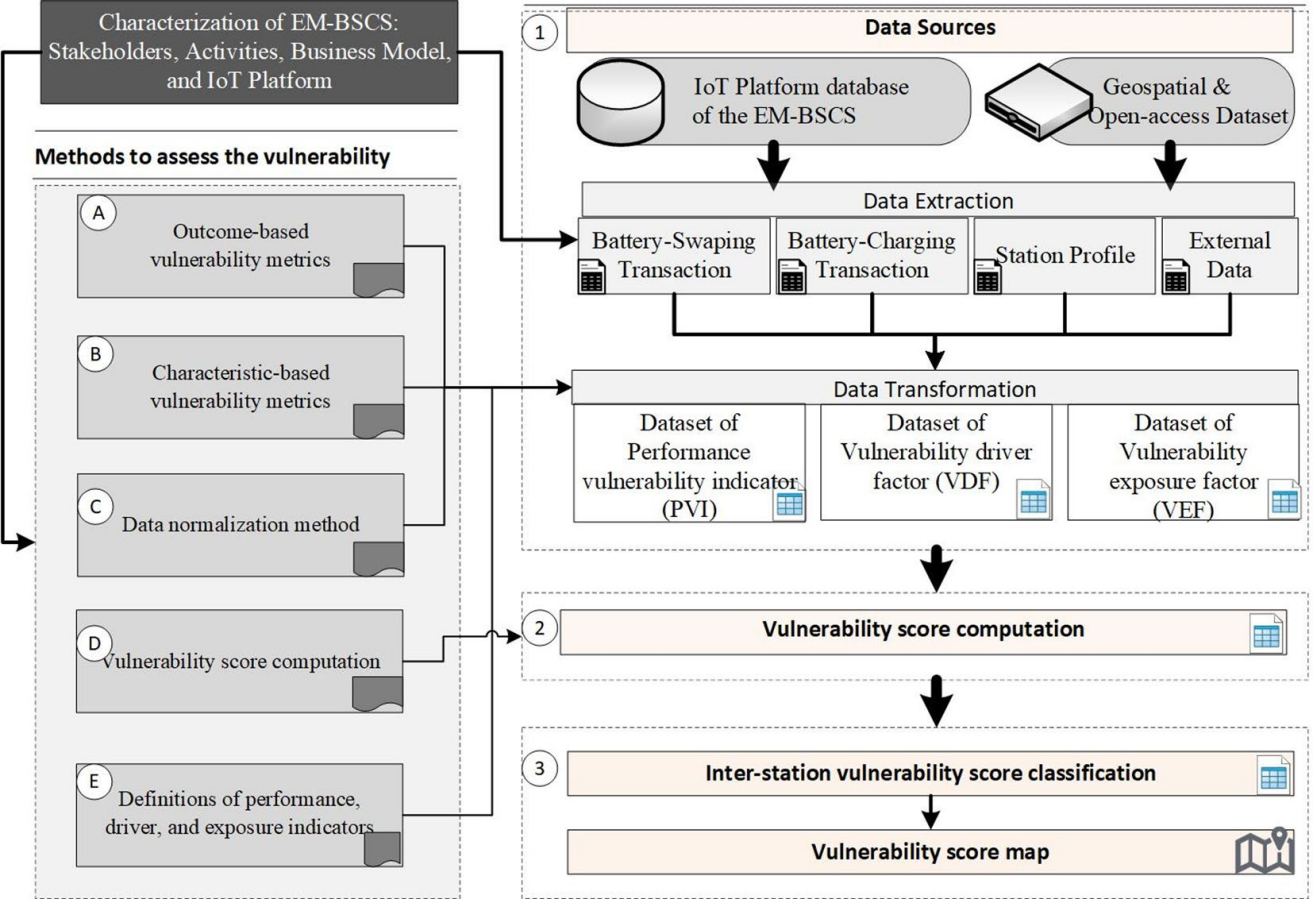
Methodology to develop vulnerability score on EM-BSCS.

The methodology is illustrated in Fig. 2. The first section (left side) elucidates the methods, including vulnerability metrics, scaling method, vulnerability scoring formulation, and description of indicators used. The second section (right side) addresses the implementation of the model in a case, including data sources, data extraction, data transformation, vulnerability score computation, and reporting and analysis.

### Proposed vulnerability concept

**Fig. 3:**
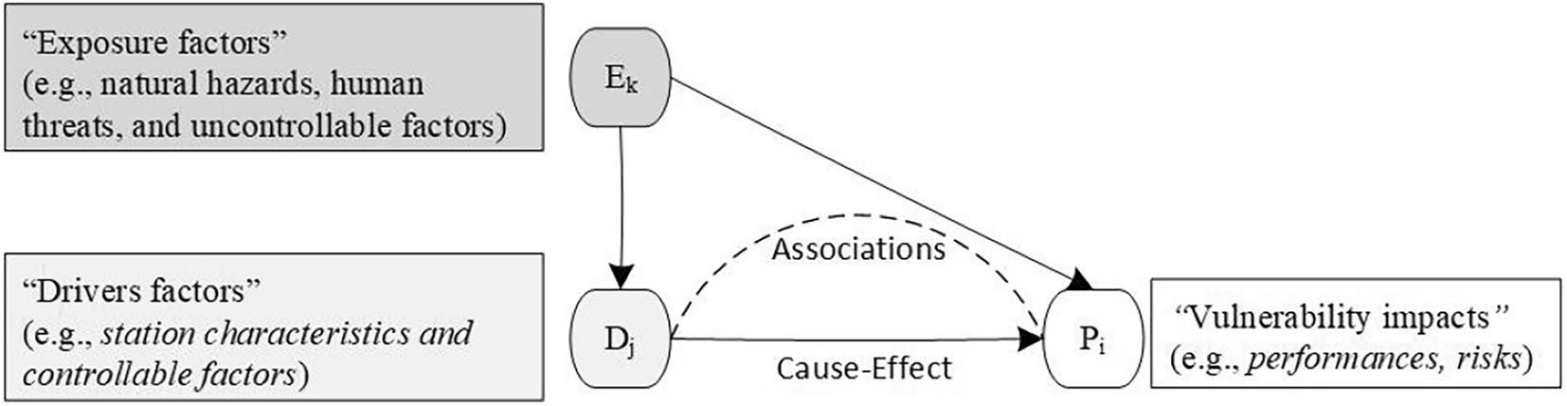
Vulnerability model in the EM-BSCS.

Based on those vulnerability concepts in the previous subsection, there are two types of vulnerability impact: risk and performance degradation. The concept of vulnerability is proposed in Fig. 3. Vulnerability refers to a condition when performance falls outside the defined bounds of the expected range, driven by internal preconditions that may be weak in resisting exposure factors, which can themselves directly exacerbate performance degradation. Additionally, the system is vulnerable while performance remains outside the defined bounds, neither improving nor recovering, but instead continuing to deteriorate. This concept adopts performance measures as vulnerability impacts or system outcomes, as it is more readily observable in big data than risk-based measures. Both the magnitude and frequency of performance degradation serve as metrics for assessing vulnerability levels.

Internal preconditions are driver factors that refer to operational characteristics of the EM-BSCS, which represent the system’s mitigation ability and may drive an increase or decrease in the vulnerability impacts. For instance, the availability of cabinets and batteries at a station enhances mitigation capability, thereby reducing performance impacts like low battery swapping throughput. Similarly, fewer customers swapping batteries at SoC levels below the ideal threshold can minimize battery degradation, leading to greater diversity in battery circulation and higher swapping throughput. Ultimately, these internal precondition factors (driver factors) reflect system conditions that are within operational control.

Exposure factors are station characteristics that represent external influences or uncontrollable factors (e.g., natural hazards, human threats) that may exploit driver factors and intensify the impact of vulnerability (performance degradation). For example, flood risk at a station might hinder mitigation capability and degrade system performance. The quantity of operational failure modes in battery swapping could increase the swapping duration time and decrease the throughput levels. Likewise, higher customer engagement in battery charging services could reduce cabinet availability for battery swapping and decrease throughput levels.

### Outcome-based vulnerability metrics

Outcome-based vulnerability metrics are measured through performance values. A system is considered vulnerable (VL = 1) at time *t* if the performance value $x_t$ falls outside the defined bounds of the expected (robustness) range (see Eq. 1). Time $t$ is the smallest period in which the performance value can be observed (e.g., hourly or daily), and $T$ is the length of the observation time (e.g., a month). The bounds, an upper bound (UB) or a lower bound (LB), serve as a determinant of whether a performance value is vulnerable or not because it falls outside the bounds established by stakeholders. For example, the company states that if the expected range of daily swapping transactions (throughput) in a station is more than 30 transactions, then the lower bound is 30, and the performance is vulnerable if the daily transaction is below the lower bound.

1 $$\begin{aligned} VL = {\left\{ \begin{array}{ll} 1, & \text {if } [(LB \ exist \ and \ x_t < LB)] \ V \ [(UB \ exist \ and \ x_t > UB)] \\ 0, & otherwise \end{array}\right. } \end{aligned}$$

The lower and upper bounds of each performance variable may be defined by stakeholders based on specific criteria or derived from the first (Q1) and third (Q3) quartiles of historical data. Alternatively, upper and lower bounds may be defined by applying one-dimensional K-means clustering^53^ to divide the data into several classes, then use a specific class’s boundaries as the upper and lower bounds. Applying clustering methods is a precautionary approach when data deviate from a normal distribution.

**Fig. 4:**
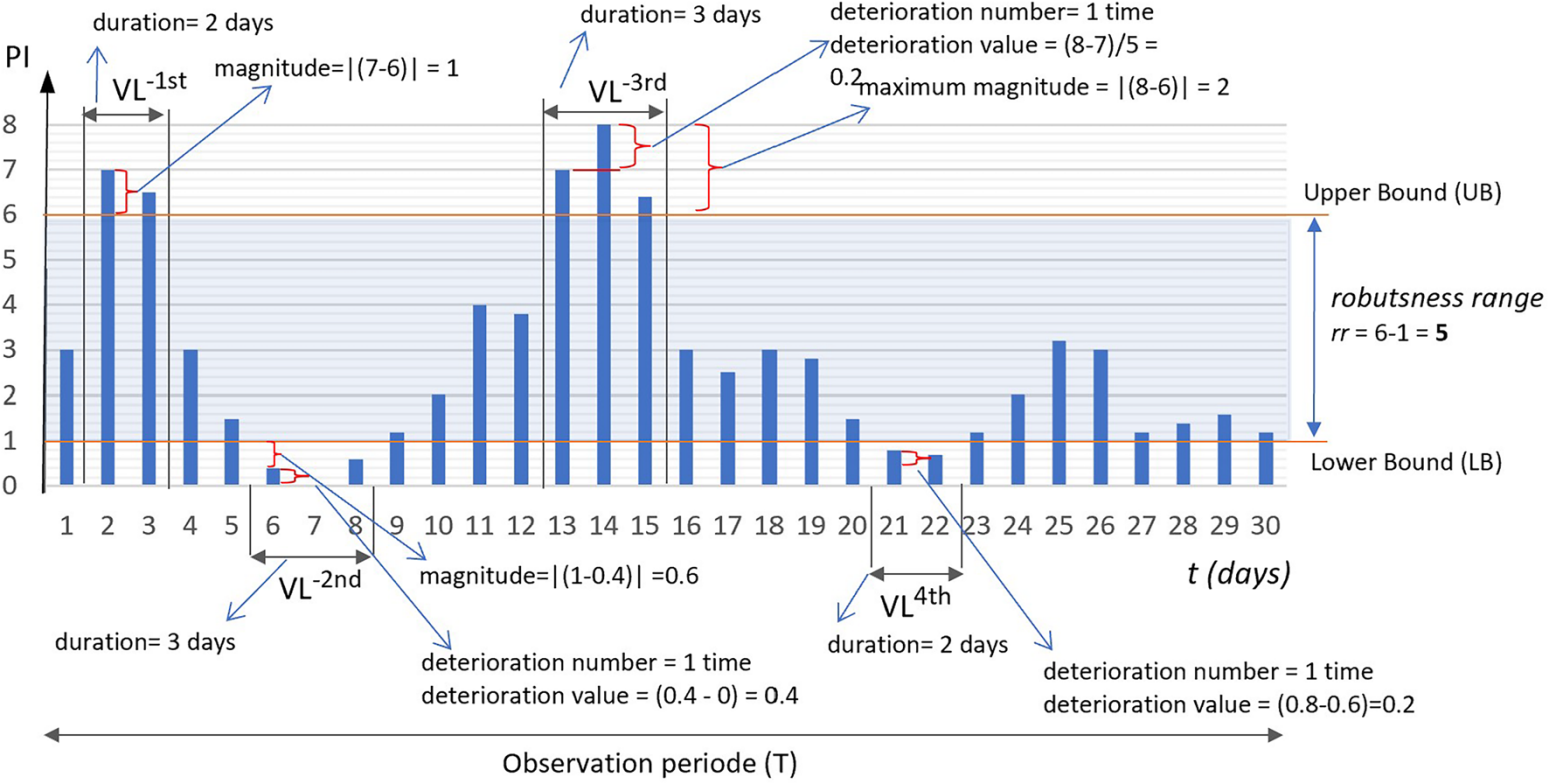
Vulnerability events for a performance indicator.

Outcome-based vulnerability metrics were adopted from Vulnerability Assessment (VULA)^49^. In contrast to VULA, this metric considers events in which system performance fails to recover and instead continues to deteriorate. The metrics include magnitude, duration, frequency, and deterioration-based metrics, as illustrated in Fig. 4. The performance value is assumed to have a lower and upper bound, where *t* is daily, and *T* is 30 days.

**Magnitude-based metrics (MVM)** quantify the total and maximum deviations (magnitude) of performance values $x_t$ at time *t* below the lower bound LB (when defined) or exceeding the upper boundU UB. It is assumed that larger deviations indicate greater vulnerability. This metric reflects the hazard level associated with vulnerability impact. The magnitude value at time *t* ( $MV_t$ ) is defined as the absolute difference between the value $x_t$ and either the upper or lower bound (see Eq. 2). The total magnitude (MVM1) represents the overall impact of performance deviations (see Eq. 3), whereas the maximum magnitude (MVM2) reflects the worst-case performance (see Eq. 4).

2 $$\begin{aligned} MV_t = {\left\{ \begin{array}{ll} |x_t-UB|, & if \ UB \, exist \ and \ x_t > UB \\ |x_t-LB|, & if \ LB \, exist \ and \ x_t < LB \\ 0, & otherwise \end{array}\right. } \end{aligned}$$

3 $$\begin{aligned} MVM1 = \sum _{t \in T} MV_{t} \end{aligned}$$

4 $$\begin{aligned} MVM2 = \max {(MV_t)} \end{aligned}$$

Figure 4 illustrates: 

$$MVM1 = 1 + 0.5 + 0.6 + 1 + 0.2 + 1 + 2 + 0.5 + 0.2 + 0.4 = 7.4$$

$$MVM2 = 2$$

**Duration-based metrics (DVM)** measure the total duration (DVM1) and the longest duration (DVM2) for which the performance value $x_t$ remains outside the specified bounds of the robustness range. The duration of each out-of-range interval, $\Delta t_{i}$, denotes the time span between two consecutive observation points t while the performance remains outside the specified bounds of the robustness range. A higher DVM indicates that the system is more fragile. The interval is calculated in Eq. (5). The total duration (DVM1) and the longest duration (DVM2) are represented in Eqs. (6) and (7), respectively.

5 $$\begin{aligned} \Delta t_{i} = t_{end,i} - t_{start,i} + 1 \end{aligned}$$

6 $$\begin{aligned} DVM1 = \sum _{i=1}^{n} \Delta t_{i} \end{aligned}$$

7 $$\begin{aligned} DVM2 = \max {\Delta t_{i}} \end{aligned}$$

Figure 4 shows that performance stays outside the bounds from t=2 to t=3 and then again from points t=6 to t=8, so the duration $\Delta t_{1}$ is 2 days and $\Delta t_{2}$ is 3 days, respectively. Then DVM1 and DVM2:

$$DVM1 = 2 + 3 + 3 + 2 = 10 \, days$$

$$DVM2 = 3 \, days$$

**The Frequency-based Metric (FVM)** counts the number of distinct episodes where the performance value lies outside the expected range boundaries during the observation period *T*. Each episode may span multiple consecutive time points but is counted as a single occurrence (see Eq. 8).

8 $$\begin{aligned} FVM1 = \sum _{t \in T} 1 (VL_{t} = 1 \, and \, VL_{t-1} = 0) \end{aligned}$$

Figure 4 illustrates that FVM1, the frequency of vulnerable conditions, occurs four times: twice below the lower bound and twice above the upper bound.

**Deterioration-based metrics (DTM)** count the condition when the performance value $x_t$ remains outside the defined robustness boundaries and shows no improvement toward recovery but instead continues to deteriorate. This metric represents quantifying the severity of vulnerability. Equation (9) defines a condition when a system deteriorates. Equation (10) denotes the magnitude of deterioration at time *t*.

9 $$\begin{aligned} \begin{aligned} DT_t&= {\left\{ \begin{array}{ll} 1, & \text {if } \left[ \begin{array}{l} x_t< LB \text { and } x_t - x_{t-1} < 0 \\ \text {or} \\ x_t> UB \text { and } x_t - x_{t-1} > 0 \end{array} \right] \\ 0, & \text {otherwise} \end{array}\right. } \\&= {\left\{ \begin{array}{ll} 1, & \text {if } \bigl ( x_t < LB \wedge x_t - x_{t-1} < 0 \bigr )\ \vee \ \bigl ( x_t > UB \wedge x_t - x_{t-1} > 0 \bigr ),\\ 0, & \text {otherwise}. \end{array}\right. } \end{aligned} \end{aligned}$$

10 $$\begin{aligned} d_t = |x_t - x_{t-1} |,\ \text {if } \left[ \begin{array}{l} x_t < LB \text { and } x_t - x_{t-1} < 0 \\ \text {or} \\ x_t > UB \text { and } x_t - x_{t-1} > 0 \end{array} \right] \end{aligned}$$

Equation (11) define the total deterioration magnitude (DTM1). DTM1 represents the accumulated magnitude by which performance continues to deviate further away from the expected range during periods of vulnerability. It is computed as the sum of absolute differences between consecutive performance values when such values lie outside the robustness range and exhibit a deteriorating trend.

11 $$\begin{aligned} \begin{aligned} DTM1&= \sum _{t=2}^{T} \Bigl [ \textbf{1}\bigl ( x_t< LB \wedge (x_t - x_{t-1})< 0 \bigr ) \cdot d_t + \textbf{1}\bigl ( x_t> UB \wedge (x_t - x_{t-1})> 0 \bigr ) \cdot d_t \Bigr ] \\&= \sum _{t=2}^{T} \Bigl [ \textbf{1}\bigl ( x_t< LB \wedge (x_t - x_{t-1}) < 0 \bigr ) \cdot |x_t - x_{t-1} |+ \textbf{1}\bigl ( x_t> UB \wedge (x_t - x_{t-1}) > 0 \bigr ) \cdot |x_t - x_{t-1} |\Bigr ]. \end{aligned} \end{aligned}$$

Equations (12) define the number of deterioration occurrences (DTM2). DTM2 quantifies how many times performance begins to deteriorate further outside the robustness range across the observation period. Each occurrence signals a new episode of vulnerability intensification.

12 $$\begin{aligned} \begin{aligned} DTM2&= \sum _{t=2}^{T} \textbf{1}\bigl ( DT_t = 1 \wedge DT_{t-1} = 0 \bigr ) \\&= \sum _{t=2}^{T} \textbf{1}\Bigl ( \bigl ( x_t< LB \wedge (x_t - x_{t-1}) < 0 \bigr ) \wedge \bigl ( DT_{t-1} = 0 \bigr ) \Bigr ) + \sum _{t=2}^{T} \textbf{1}\Bigl ( \bigl ( x_t> UB \wedge (x_t - x_{t-1}) > 0 \bigr ) \wedge \bigl ( DT_{t-1} = 0 \bigr ) \Bigr ) \end{aligned} \end{aligned}$$

Figure 4 illustrates that DTM2 occurs three times: twice below the lower bound and once above the upper bound.

### Characteristic-based vulnerability metrics

System characteristics encompass internal preconditions (driver factors) and external exposure factors, both potentially influencing system vulnerability as defined in the model framework. Internal preconditions include attributes such as the number of available cabinets, the distance to the nearest neighboring station, the daily number of customers swapping batteries below the standard state-of-charge (SoC) threshold, and the number of users utilizing the station for private battery charging. In contrast, external exposure factors such as operational disruptions and environmental hazards, e.g., flooding.

Characteristic-based vulnerability metrics are computed via two methods: median absolute deviation (MAD) and static feature value ( $x_{T}$ ). Static feature ( $x_{T}$ ) is a value of an attribute from the driver or exposure factor that remains constant over the observation period. Examples of static features include the number of cabinets, distance to the nearest station, flood hazard indices, and monthly disruption counts. The values distinguish station profiles and potentially influence performance.

Conversely, process-related variables such as battery swap time, private charging duration, and customer inter-arrival times are evaluated using Median Absolute Deviation (MAD), as they typically exhibit temporal variation. MAD (Eq. 13) is preferred over the mean due to its robustness to non-normal distributions, making it more suitable for real-world data. It captures the variability in characteristic indicators over time, where higher variability may reflect a greater level of system vulnerability.

13 $$\begin{aligned} MAD = Median(|x - median(X)|) \end{aligned}$$

### Scaling method

The scaling (normalization) method is performed to reconcile differences in scale and units across variables. A min–max scaling approach is used to map values into the [0,1] interval, with scaling direction determined by the metric orientation-either larger-is-more-vulnerable (LV), where higher values imply greater vulnerability, or smaller-is-more-vulnerable (SV), where lower values indicate higher vulnerability. As shown in Eq. (14), this ensures that normalized metrics near 1 correspond to high vulnerability levels.

14 $$\begin{aligned} MinMaxScale(x) = {\left\{ \begin{array}{ll} \frac{x-min[X]}{max[X]-min[X]}, if \, LV \\ \frac{max[X]-x}{max[X]-min[X]}, if \, SV \end{array}\right. } \end{aligned}$$

### Vulnerability scoring formulation

The previous section established metrics for outcome vulnerability (i.e., performance) employing MVM, DVM, FVM, and DTM, and characteristics vulnerability using either MAD values or static feature values. The next section describes how these metrics are formulated to derive vulnerability scores for each battery swapping and charging station.

Consider a performance indicator used as an outcome-based vulnerability attribute. The performance indicator values are used to calculate vulnerability using outcome-based metrics such as MVM, DVM, FVM, and DTM, which are aggregated into the Performance Vulnerability Indicator (PVI). The outcome-based vulnerability score (OVIs) for a station *s* is computed as the average of the PVI metrics (Eq. 15a), where each $PVI_{i,s}$ represents the aggregated values of the MVM, DVM, FVM, and DTM metrics for $PVI_{i}$ at station *s* (Eq. 15b).

Similarly, let a driver factor and an exposure factor represent attributes of characteristic-based vulnerability. The characteristic-based vulnerability score (CVI) for a station *s* is calculated as the sum of the values from the vulnerability driver factor metric (VDF) and the vulnerability exposure factor metric (VEF) (Eq. 16a). The $VDF_{s}$ is defined as the average of the vulnerability driver factors at a station *s* (Eq. 16b), while the $VEF_{s}$ is computed as the average of the vulnerability exposure factors at a station *s* (Eq. 16c).

Finally, the vulnerability score for a station (Eq. 17) is obtained by aggregating the OVI_s_ and CVI_s_, where the CVI_s_ comprise the summed values of VDF_s_ and VEF_s_ (Table 1).

15a $$\begin{aligned} OVI_{s}&= \sum _{i=1}^{I} \frac{PVI_{i,s}}{I}, \forall \, s \in S \end{aligned}$$

15b $$\begin{aligned} PVI_{i,s}&= (MVM1+MVM2+DVM1+DVM2+FVM1+DTM1+DTM2)/7 , \forall \, i \in I\, , \, s \in S \end{aligned}$$

16a $$\begin{aligned} CVI_{s}&= VDF_{s} + VEF_{s}  , \forall \, s \in S \end{aligned}$$

16b $$\begin{aligned} VDF_{s}&= \sum _{j=1}^{J} \frac{VDF_{j,s}}{J}, \forall \, s \in S \end{aligned}$$

16c $$\begin{aligned} VEF_{s}&= \sum _{k=1}^{K} \frac{VEF_{k,s}}{K}, \forall \, s \in S \end{aligned}$$

17 $$\begin{aligned} \begin{aligned} VI_{s}&= OVI_{s} + CVI_{s}, \forall \, s \in S \\&= OVI_{s} + VDF_{s} + VEF_{s} , \forall \, s \in S \\&= \sum _{i=1}^{I} \frac{PVI_{i,s}}{I} + \sum _{j=1}^{J} \frac{VDF_{j,s}}{J} + \sum _{k=1}^{K} \frac{VEF_{k,s}}{K} , \forall \, s \in S \end{aligned} \end{aligned}$$

**Table 1:** Notation and Description.

Notation	Description
*VIs*	Vulnerability score at a station *s*
*OVIs*	Score of outcome-based vulnerability at a station *s*
*PVI* *i*, *s*	Score of performance vulnerability indicator *i* at a station *s*
*CVIs*	Score of characteristic-based vulnerability at a station *s*
$VDF_{s}$	Score of vulnerability driver factor at a station *s*
$VEF_{s}$	Score of vulnerability exposure factor at a station *s*
*s*	Index of EM-BSCS stations, $1,2,\dots ,S$
*i*	Index of performance indicator, $1,2,\dots ,I$
*j*	Index of driver factors, $1,2,\dots ,J$
*k*	Index of disturbance factors, $1,2,\dots ,K$

### Performance vulnerability indicator (PVI), vulnerability driver factor (VDF) and vulnerability exposure factor (VEF)

A performance vulnerability indicator (PVI) refers to a set of system performance variables used to characterize vulnerability conditions, particularly when the performance values fall outside the expected or acceptable range. Outcome-based vulnerability metrics are applied to PVI to quantitatively assess the system’s level of vulnerability.

As outlined in the subsection Proposed Vulnerability Concept, two key terms are introduced: internal preconditions (driver factors) and exposure factors. In the following discussions, these terms will be referred to as the Vulnerability Driver Factor (VDF) and the Vulnerability Exposure Factor (VEF), respectively. Metrics such as MAD and static features (*x*_*T*_) are applied to the VDF and VEF, as will be elaborated in the following sections.

This section introduces some indicators of PVI, VDF, and VEF, in Tables 2, 3, and 4. The indicators or factors are adopted from prior studies such as those on manufacturing systems, logistics, and warehousing^35,40,45,54^, battery charging infrastructure^20,38,55,56^, battery storage systems^57^, and the British Standard - Maintenance Key Performance Indicators^58^. The selection of indicators and factors introduced in Tables 2 through 4 is limited by the scope of attributes available in the dataset obtained from an EM-BSCS service provider in Jakarta^59^.

Table 2 presents three examples of performance vulnerability indicators (PVIi), one of which is battery swapping throughput ( $PVI_{1}$ ). Throughput may serve as a basis for station closure decisions when the value is critically low^55^. Throughput data, combined with the state of charge (SoC) of swapped batteries, can be used to estimate daily electricity consumption (kWh), which may design incentive schemes for users swapping batteries above a specific SoC threshold. Additionally, throughput can be used to calculate station occupancy^38^. Similarly, the second indicator ( $PVI_{2}$ ) may capture battery utilization diversity, measured as the ratio of unique batteries ( $PVI_{2}$ ) to swapping throughput ( $PVI_{1}$ ). A higher ratio suggests lower vulnerability by reducing the risk of accelerated battery degradation.

Characteristic-based vulnerability variables include several driver and exposure factors, as presented in Tables 3 and 4. These factors may vary depending on system context and data availability. The state of charge (SoC) is an example of a driver factor, as frequent battery swaps at low SoC levels can reduce battery availability and accelerate degradation, thereby increasing EM-BSCS vulnerability. The median absolute deviation (MAD) of the SoC at each station is used as input for vulnerability scoring. Meanwhile, variables such as flood index and distance to the nearest station are considered static features ( $x_{T}$ ), as they remain relatively stable over observation periods. Definitions and measurement methods for all factors are provided in Tables 3 and 4.

**Table 2:** The performance vulnerability indicators.

Code	Indicator	Description and metric	Reference
PVI_1_	Battery-swapping throughput	The total number of battery-swapping transactions per day.	Priyandari^60^, Helmus^55^
PVI_2_	Unique batteries swapped.	The total number of unique batteries swapped per day.	Helmus^55^
PVI_3_	Battery-swapping customers	The total number of consumers engaged in daily battery swapping.	Helmus^55^

**Table 3:** The vulnerability driver factors.

Code	Factor	Description and metric	Reference
VDF_1_	State of charge (SoC)	The State of Charge (SoC), reflecting a battery’s remaining energy, varies substantially among incoming batteries and impacts vulnerability. Its variability is measured by MAD, where higher MAD values imply greater vulnerability.	Emeric^57^
VDF_2_	SoC below 15%	This attribute is the total number of batteries swapped with an initial State of Charge (SoC) below 15% over the observation period *T*. The 15% threshold is determined by the EM-BSCS operator. This indicator is treated as a static feature $x_{T}$, where higher values of $x_{T}$ signify increased operational vulnerability.	Emeric^57^
VDF_3_	Customer inter-arrival time.	Customer inter-arrival time is defined as the interval between successive battery swap customers. This variability is quantified via MAD.	.
VDF_4_	Swap completion time	Swap completion time measures the duration from compartment opening to closure during a battery exchange. Variability in this time, influenced by both technical (e.g., signal strength, battery condition) and behavioral factors, is quantified using MAD.	^55^
VDF_5_	Charging duration	Charging duration is the time a customer spends recharging a private battery, influenced by initial SoC, target SoC, and other factors. This variability is quantified via MAD.	Helmus^55^
VDF_6_	The SoC of the recharged battery	This attribute captures the initial SoC of batteries entering recharging services, and it is assessed via MAD.	^55^
VDF_7_	Battery charging throughput	Battery charging throughput is the daily number of batteries recharged (charging service). This attribute is classified as a driver factor rather than a performance indicator because charging remains a secondary service in EM-BSCS with relatively low transaction volumes. This attribute is assessed using a static feature $x_{T}$; greater charging throughput indicates higher operational vulnerability.	.
VDF_8_	The distance between station	Travel distance from neighboring battery swapping stations is evaluated as a static feature $x_{T}$; greater distances indicate higher operational vulnerability.	.
VDF_9_	Activated compartment	The number of active compartments at a station, assessed via the static feature $x_{T}$; lower $x_{T}$ values signify greater operational vulnerability.	Prianjani^61^

**Table 4:** The vulnerability exposure factors.

Code	Factor	Description and metric	Reference
VEF_1_	Operational disruption	Operational disruption refers to any incident or condition that interrupts, delays, or impairs the normal battery swapping and charging processes at a station. The total number of disruptions over the observation period *T* is quantified as a static feature, $x_{T}$.	Sharma^45^
VEF_2_	Flood potential level	An index quantifying the flood hazard level in the vicinity of the station.	Sharma^45^, Dev^35^, Kamble^54^

**Fig. 5:**
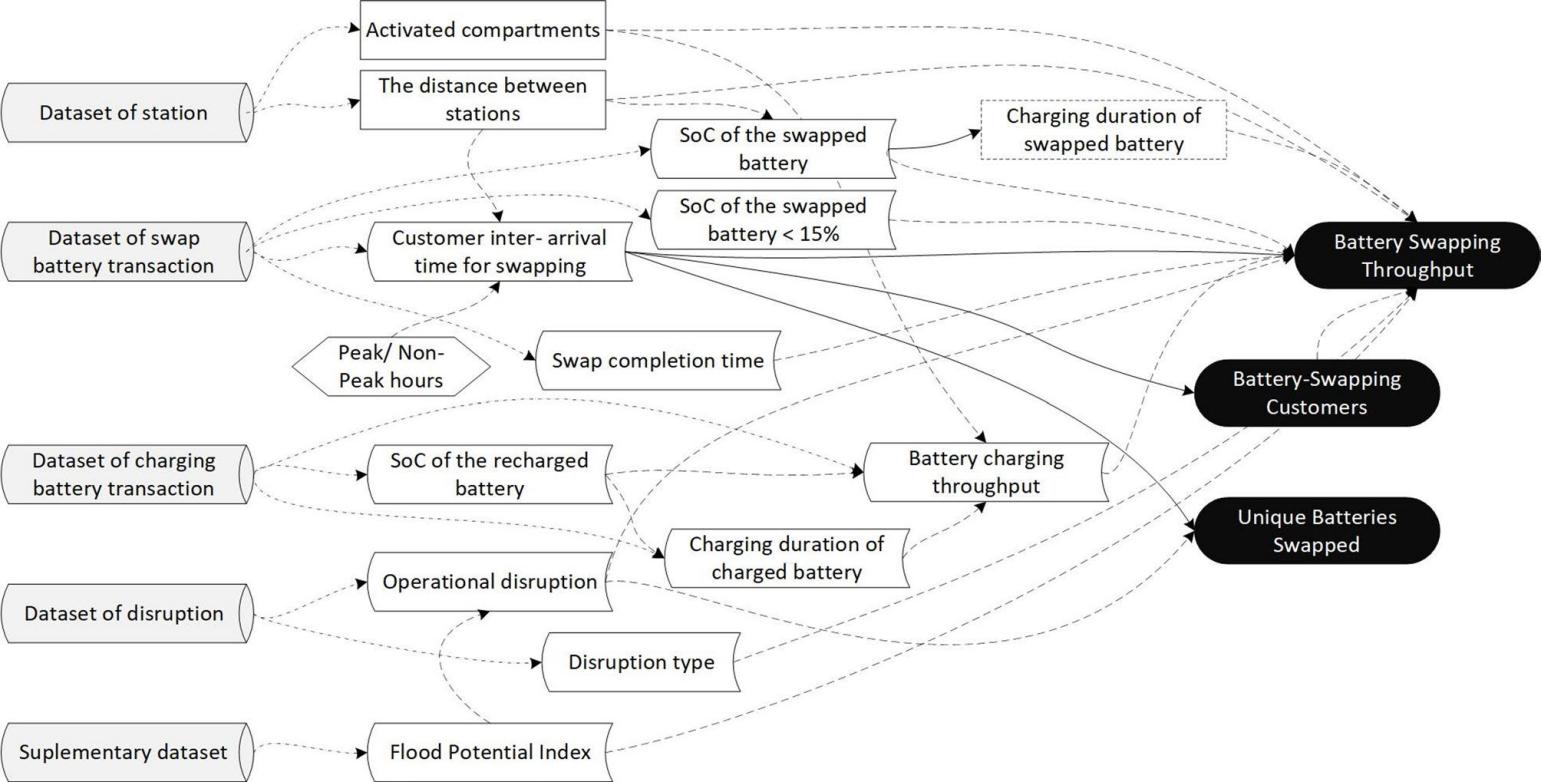
The relation scheme of PVI, VDF and VEF.

Figure 5 illustrates a relationship between the driver and exposure factors, which are assumed to affect the outcomes of the battery swapping and charging operations.

## Results

These sections describe the implementation of the vulnerability scoring model. The first part details the data sources, extraction procedures, and transformation into a structured dataset for calculating operational vulnerability scores. The final part explains the classification of vulnerability scores, the spatial mapping of station vulnerabilities, and the subsequent analysis, interpretation, and operational implications for the EM-BSCS.

### Data sources and processing

The data source originated from the IoT platform database of the EM-BSCS operator^59^. The extracted data includes the profile of EM-BSCS, battery swapping transactions, charging transactions, and operational disruption records. The dataset covers 34 stations in Jakarta, with transaction data spanning October to December 2023. Additional data sources include the Jakarta flood hazard index from Satu Data Jakarta^62^ and the Rupa Bumi Indonesia (RBI) geospatial for Jakarta^63^.

**Fig. 6:**
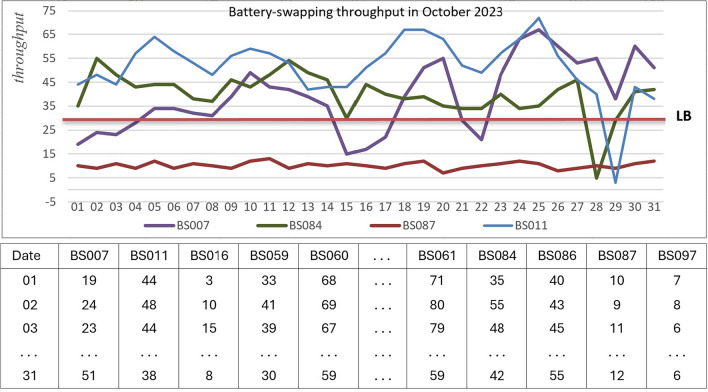
Batteries swapping throughput in October 2023.

Data extraction and transformation processes were performed to generate the required datasets for indicators and factors in Tables 2, 3, and 4. Figure 6 shows an example of a dataset, the throughput of swapping transactions in October 2023 for some stations. The figure illustrates that the throughputs fluctuate and sometimes fall below the lower bound (LB = 30 transactions per day, as defined by the EM-BSCS company). The detailed dataset is provided in the Appendices.

### Vulnerability score calculation

Outcome-based vulnerability metrics (MVM, DVM, FVM, and DTM) were applied to performance vulnerability indicators (PVI), with metric values normalized via min-max scaling. Results for October, November, and December are presented in Tables A.1, B.1, and C.1 for PVI_1_–battery swapping throughput (showing ten sample entries; complete data is available on GitHub).

Conversely, characteristic-based vulnerability metrics (MAD or static value *x*_*T*_) were used for attributes of driver factors (VDF_1_ to VDF_9_) and exposure factors (VEF_1_ and VEF_2_) for each station *s*, summarized in Tables A.2, B.2, and C.2. These tables also report scores for PVI_2_–unique batteries swapped and PVI_3_–battery swapping customers. Using Eqs. (15a) to (17), we computed the OVIs, VDFs, VEFs, and ultimately the VIs (vulnerability score) for each station *s*. Table 5 resumes scores of the OVIs, VDFs, VEFs, and VIs for the ten locations (complete data for 34 locations in Jakarta is available on GitHub). Table D.1 presents a summary of the vulnerability scores across 34 locations for the months of October, November, and December 2023.

**Table 5:** The vulnerability scores (VIs).

Station	October				November				December			
	OVIs	VDFs	VEFs	VIs	OVIs	VDFs	VEFs	VIs	OVIs	VDFs	VEFs	VIs
BS007	0.23	0.33	0.02	0.57	0.19	0.38	0.02	0.59	0.20	0.40	0.14	0.74
BS011	0.15	0.41	0.36	0.91	0.05	0.47	0.23	0.75	0.00	0.36	0.25	0.61
BS016	0.71	0.26	0.00	0.97	0.71	0.19	0.00	0.90	0.70	0.21	0.00	0.92
BS059	0.16	0.38	0.00	0.54	0.09	0.35	0.00	0.44	0.24	0.31	0.04	0.59
BS060	0.00	0.40	0.27	0.67	0.00	0.31	0.23	0.54	0.00	0.31	0.23	0.55
...	...	...	...	...	...	...	...	...	...			
BS061	0.00	0.21	0.09	0.30	0.00	0.24	0.07	0.31	0.00	0.25	0.02	0.26
BS084	0.16	0.31	0.38	0.84	0.43	0.24	0.23	0.90	0.60	0.26	0.25	1.11
BS086	0.09	0.34	0.25	0.68	0.08	0.38	0.27	0.72	0.00	0.30	0.23	0.54
BS087	0.64	0.40	0.00	1.04	0.66	0.32	0.00	0.98	0.69	0.27	0.00	0.96
BS097	0.78	0.29	0.50	1.57	0.74	0.34	0.07	1.15	0.51	0.30	0.05	0.86

## Discussion

**Fig. 7:**
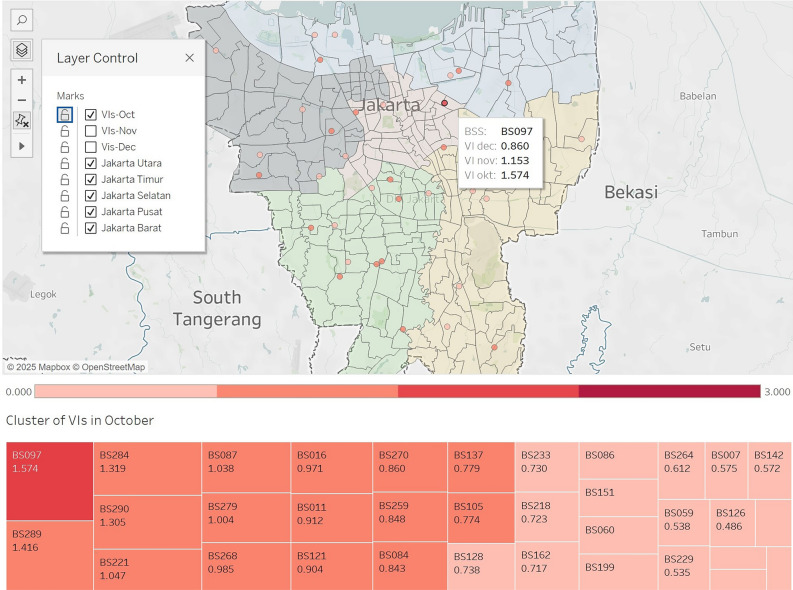
Vulnerability scores in October at the EM-BSCS stations. The map was generated using Tableau Desktop Professional Edition version 2024.2.2 (2024.24.0807.0327) (research license) (https://www.tableau.com/products/desktop) with the geospatial data of Rupa Bumi Indonesia (RBI) from Badan Informasi Geospasial (https://tanahair.indonesia.go.id/portal-web/unduh/rbi-wilayah)^63^.

The vulnerability scores range from 0 to 3 and are classified into four categories: not vulnerable, potentially vulnerable, moderately vulnerable, and vulnerable, as shown in Table 6. Based on the calculated vulnerability scores (VIs) for 34 sample stations in Jakarta, the majority fall into the not vulnerable and potentially vulnerable categories. Figure 7 illustrates the spatial distribution of stations according to these vulnerability levels for October 2023. Six stations are identified as moderately vulnerable, distributed across all areas of Jakarta. One station, BS097 (VIs=1.574), was identified as vulnerable, which is in Jakarta Pusat.

**Table 6:** The Vulnerability cluster based on the VI scores of the stations.

Level	Scale	Clasification	October	November	December
1	VI < 0.75	not vulnerable	18	17	18
2	0.75 <= VI < 1	potentially vulnerable	9	10	9
3	1 <= VI < 1.5	moderately vulnerable	6	7	6
4	VI >= 1.5	vulnerable	1	0	1

**Fig. 8:**
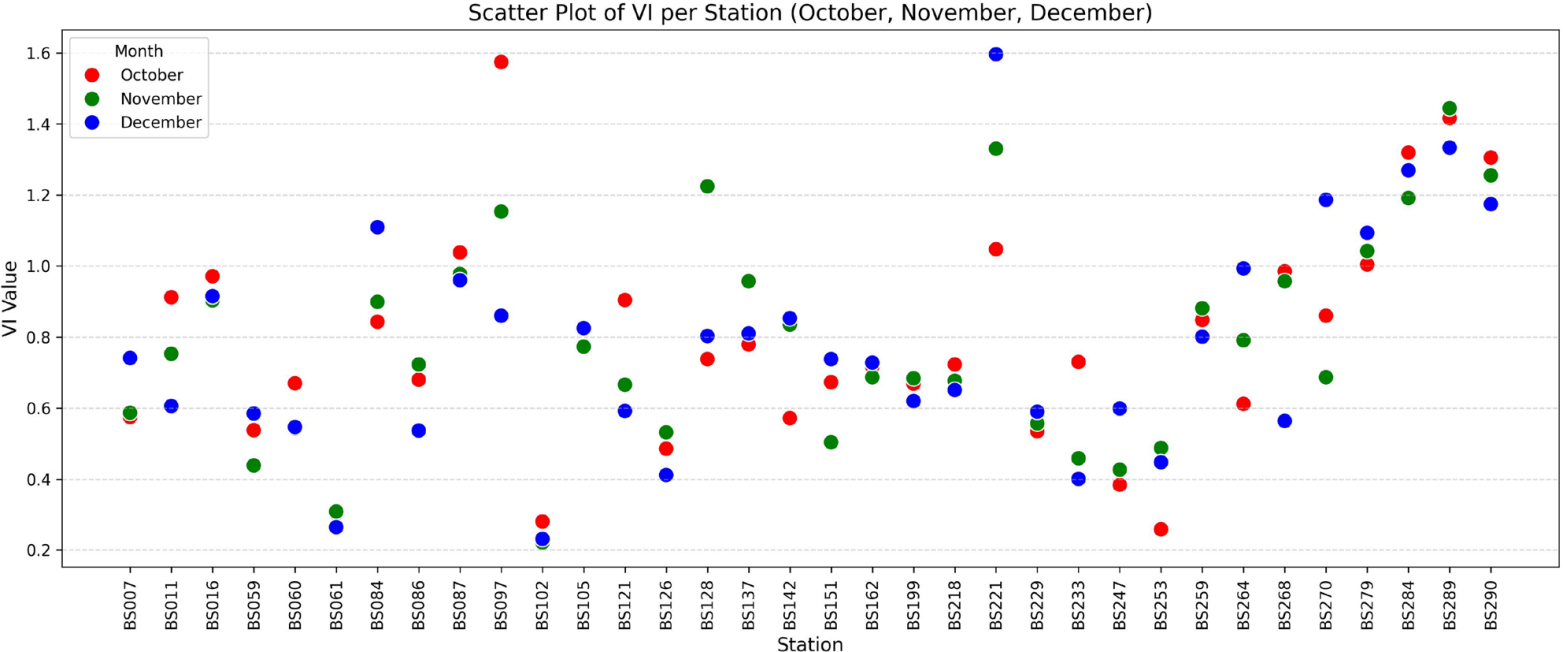
The vulnerability scores from October to December.

Figure 8 reveals that there are high deviations in vulnerability metrics for several stations compared to others. For example, stations BS097, BS121, and BS233 experienced a significant decrease in vulnerability scores from October to December. The station BS097 has VIs 1.574 in October, then 1.153 and 0.860 in November and December, respectively (see Table 5 in subsection result or in Table D in Appendices). In contrast, station BS221 exhibited a substantial increase in its vulnerability score, while the VIs scores are 1.047, 1.330, and 1.596 from October to December. Furthermore, some stations showed fluctuations; for example, BS128 recorded a low score in October (0.738), a sharp increase in November (1.224), and a significant decrease again in December (0.803).

Visually, Fig. 8 indicates differences in vulnerability levels among stations. This observation is supported by a one-way ANOVA test, which yielded a p-value of $3.93 \times 10^{-18}$, indicating significant variance differences across stations. Subsequent Tukey HSD analysis reveals that stations with high VIs scores (BS097, BS221, BS284, BS289, BS290) differ significantly from many other stations with lower scores (such as BS007, BS061, and others). These differences are primarily driven by (i) variations in operational performance indicators (outcome-based vulnerability), such as battery-swapping throughput, customer activity, and unique battery usage; (ii) heterogeneity in station-specific operational characteristics (driver factors), including state of charge (SoC), swap completion time, and customer inter-arrival patterns; and (iii) differing exposure to external disturbances (exposure factors), such as flood potential and frequency of operational disruptions. For instance, BS097 exhibited high deviations in performance metrics in October, while other stations showed lower variability. These factors, when integrated into the vulnerability score, reveal patterns of potential causal influence, where specific internal attributes and external conditions co-occur with performance degradation. While causal relationships are not formally established in this study, the strong empirical associations presented in the results highlight operational signals that can serve as proxies for root causes. This insight enables early detection and targeted interventions at the station level.

**Fig. 9:**
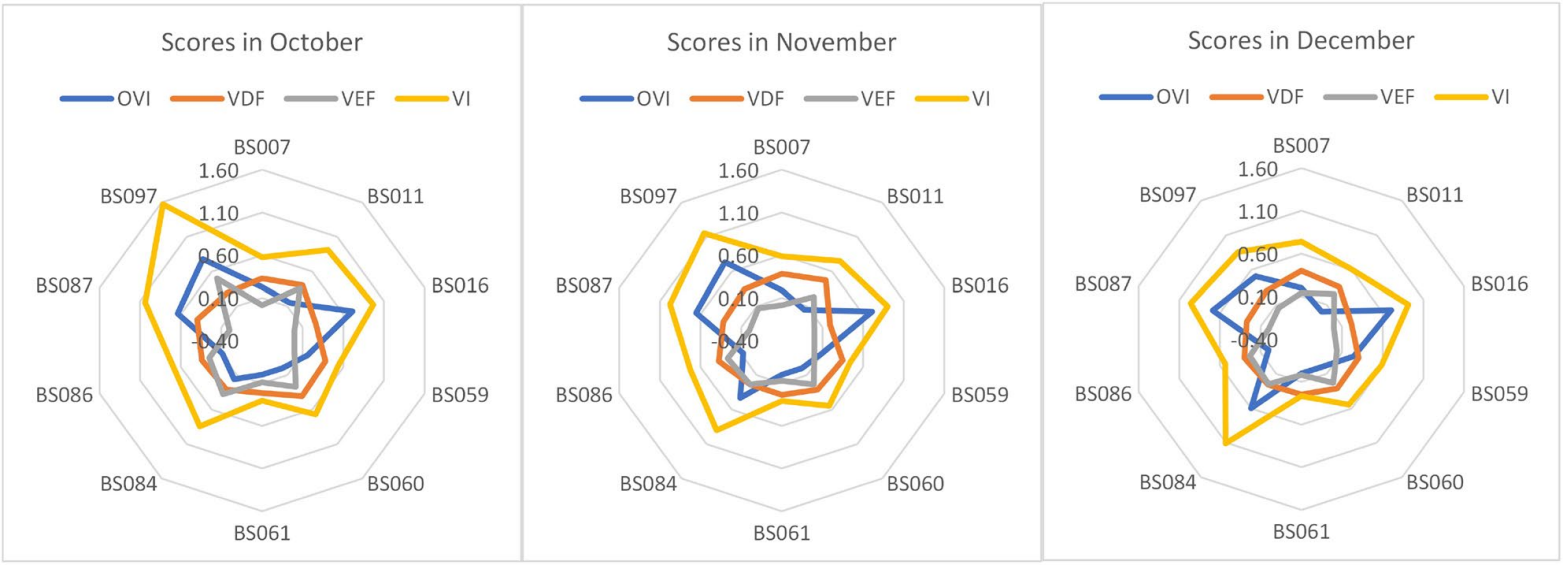
Relation of VIs with outcome and characteristic metrics.

**Fig. 10:**
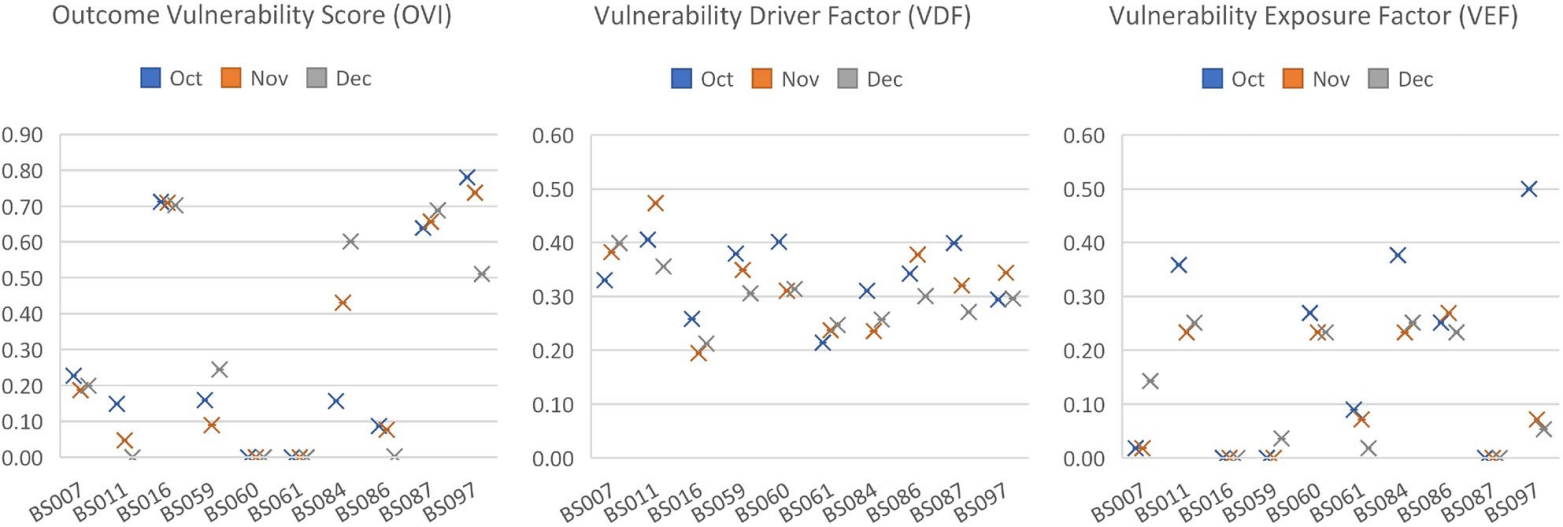
Pattern of outcome and characteristic metrics from October to December.

The spider chart in Fig. 9 shows the pattern of vulnerability scores (VIs), the value of OVIs, VDFs, and VEFs from October to December. The OVIs value appears to have a higher variation pattern than VDFs and VEFs, and it seems that changes in the OVIs pattern drive changes in the VIs value. For example, the high VIs values for BS097 in October are primarily driven by their respective OVIs profiles. This is further supported by Fig. 10, which shows that OVIs values tend to exhibit greater variation across the stations compared to the driver factor (VDFs) and exposure factor (VEFs) metrics. This finding also suggests that the EM-BSCS operator may tend to standardize station setups, such as maintaining similar numbers of active batteries across stations, even when battery-swapping transaction volumes differ.

**Fig. 11:**
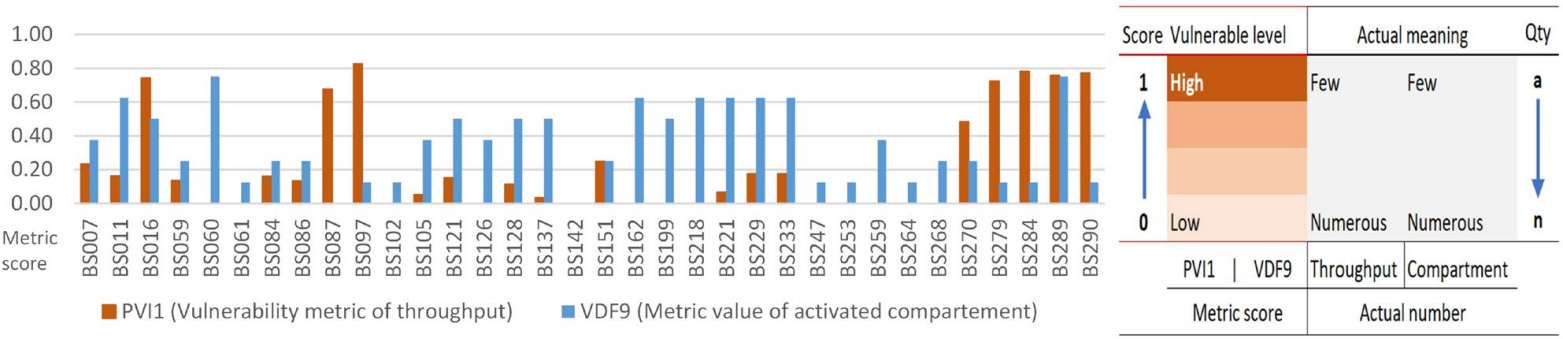
Pattern of PVI1 and VDF9.

For a more detailed analysis, Fig. 11 illustrates the relationship between values of PVI_1_ (throughput vulnerability) and VDF_9_ (the number of active compartments). Both values range from 0 to 1, where values closer to 1 indicate higher vulnerability. A high VDF_9_ corresponds to a small actual number of active compartments, so this was normalized using the “smaller is more vulnerable” rule-fewer active compartments yield a higher potential vulnerability. In contrast, throughput values are processed using outcome-based metrics reflecting the “larger is more vulnerable” rule. The outcome-based metrics capture performance deviation based on magnitude, duration, frequency, and deterioration. BS087 and BS097 stations exhibit a high PVI_1_ score (low throughput) but a low VDF_9_ (numerous active compartments), suggesting a mismatch that may imply operational inefficiency. Conversely, BS162 to BS233 stations show low PVI_1_ (high throughput) but high VDF_9_ (few active compartments), indicating a potential shortage of active compartments. These findings emphasize the necessity of aligning battery quantity control with actual system performance.

As reviewed in the literature, Table 7 summarizes the differences in attributes between the VULA^49^ model and the outcome and characteristic-based vulnerability metric (OC-Vul) model in this study. This model excludes MPI2 (mean magnitude of deviation from expected range), TPI2 (average duration of vulnerability periods), and TPI5 (fraction of time outside the expected range). In contrast, the VULA model does not include DTM1 and DTM2, which capture the deterioration concept, highlighting the system’s inability to recover or improve (lack of resilience)^46^. Moreover, VULA does not account for internal preconditions (termed vulnerability driver factors in this model) or external exposure factors that may influence performance. These differences in attributes lead to variations in the resulting vulnerability scores between the two models.

**Table 7:** Mapping of model components to performance and characteristic attributes.

Model	Outcome (performance)										Characteristic	
	1	2	3	4	5	6	7	8	9	10	11	12
VULA	MPI1	MPI3	MPI2	TPI1	TPI2	TPI4	TPI3	TPI5	-	-	-	-
OC-Vul	MVM1	MVM2	-	DVM1	-	DVM2	FVM1	-	DTM2	DTM1	VDF	VEF

Compared with Nowakowski^48^, the current model is more pertinent for quantitative analyses using IoT Platform datasets than relying on expert opinion quantification. The OC-Vul model, integrating outcome in performance and characteristics (driver and exposure factors), is more relevant as a monitoring tool for the EM-BSCS than relying solely on performance indicators. A station may exhibit better performance over some periods, but it is still more vulnerable because of other factors.

This layered integration of outcome and characteristic metrics, tailored for EM-BSCS, is a novel contribution that moves beyond prior vulnerability assessments in electric vehicle charging studies. By operationalizing both internal inefficiencies and contextual risks from real-world IoT data, the model offers new insights for proactive station-level resilience management.

## Conclusion

This study developed the vulnerability assessment model (OC-Vul), which integrates outcome-based and characteristic-based measurements. The outcome-based approach prioritizes performance indicators over risk indicators due to their greater suitability for quantitative analysis using data from the EM-BSCS IoT platform. Meanwhile, the characteristic-based approach relies on driver factors (internal preconditions) and exposure factors that can influence system performance.

A key strength of the model lies in its ability to identify the link between vulnerability impacts on performance and suboptimal station infrastructure settings, as demonstrated by the relationship between throughput vulnerability and the number of activated cabinets or batteries. Additionally, the model supports monitoring the effects of operational disruptions on vulnerability. This insight can be visualized in a dashboard system.

Vulnerability scores are categorized into four levels: not vulnerable, potentially vulnerable, moderately vulnerable, and vulnerable. This classification facilitates geospatial monitoring through the EM-BSCS IoT dashboard. Moreover, the inclusion of deterioration metrics in performance assessments adds value by detecting stations prone to declining performance.

Nonetheless, the model has limitations, as it currently assumes equal importance among all indicators, including performance metrics, drivers, and triggers. Ideally, weighting these indicators should be incorporated into the vulnerability scoring process. Future development could address this through weighting techniques such as the Best-Worst Method (BWM) or DEMATEL^64,65^, integrating stakeholder perspectives.

Although this study focused on a limited set of performance indicators, drivers, and exposure factors based on available data, the proposed vulnerability scoring framework is flexible and extensible. The model allows for the integration of additional indicators such as energy consumption, operational costs, revenue, and response latency.

Another limitation is that the current model does not attempt to infer causality in a statistical or structural sense. All vulnerability metrics are interpreted based on co-occurrence and observed variability. Future research should consider the integration of causal inference methods, such as structural equation modeling, causal graphs, or counterfactual analysis, to formally identify and validate cause-effect relationships between operational characteristics and vulnerability outcomes. This would significantly enhance the decision-making value of the model, shifting from diagnostic to prescriptive analytics^66,67^.

## Supplementary Information

Supplementary Information.

## Data Availability

The datasets for calculating the vulnerability score are available in the Appendices section or at the following URL https://github.com/priyandari/embscs-dataset.
