# Supplementary material for: Vulnerability assessment model integrating outcome and characteristic-based metrics for electric motorcycle battery swapping and charging stations
Source: Sci Rep. 2025 Oct 21;15:36613. doi: 10.1038/s41598-025-20325-x (PMC12540840; doi:10.1038/s41598-025-20325-x)
Supplement: Supplementary file 1 — Supplementary Information. [file 41598_2025_20325_MOESM1_ESM.pdf]

Appendices

Table A.1. Value of the PVI1 metric for October 2023

	Outcome-vulnerability metrics							Outcome-vulnerability metrics (scaled)							
Station	MVM1	MVM2	DVM1	DVM2	FVM1	DTM2	DTM1	MVM1	MVM2	DVM1	DVM2	FVM1	DTM2	DTM1	PVI1
BS007	72	15	9	4	3	2	9	0.08	0.50	0.29	0.13	0.38	0.12	0.15	0.23
BS011	27	27	1	1	1	0	0	0.03	0.90	0.03	0.03	0.13	0.00	0.00	0.16
BS016	564	28	31	31	1	12	51	0.61	0.93	1.00	1.00	0.13	0.71	0.82	0.74
BS059	16	8	4	1	4	0	0	0.02	0.27	0.13	0.03	0.50	0.00	0.00	0.14
BS060	0	0	0	0	0	0	0	0.00	0.00	0.00	0.00	0.00	0.00	0.00	0.00
. . .	..	..	..	..	..	..	..	..	...	...	...	...	...	...	...
BS061	0	0	0	0	0	0	0	0.00	0.00	0.00	0.00	0.00	0.00	0.00	0.00
BS084	26	25	2	2	1	0	0	0.03	0.83	0.06	0.06	0.13	0.00	0.00	0.16
BS086	11	9	3	2	2	1	8	0.01	0.30	0.10	0.06	0.25	0.06	0.13	0.13
BS087	613	23	31	31	1	13	25	0.66	0.77	1.00	1.00	0.13	0.76	0.40	0.67
BS097	706	30	31	31	1	16	59	0.76	1.00	1.00	1.00	0.13	0.94	0.95	0.83

Table A.2. Value of PVI, VDF, VEF, and VI for October 2023

	Performances			OVI	Drivers									VDF	Exposures		VEF	VI
Station	PVI1	PVI2	PVI3	.	VDF1	VDF2	VDF3	VDF4	VDF5	VDF6	VDF7	VDF8	VDF9	.	VEF1	VEF2	.	.
BS007	0.23	0.20	0.24	0.23	0.16	0.34	0.11	0.86	0.26	0.24	0.34	0.29	0.38	0.33	0.04	0.00	0.02	0.57
BS011	0.16	0.15	0.14	0.15	0.21	0.47	0.10	0.82	0.23	0.58	0.61	0.00	0.63	0.41	0.25	0.47	0.36	0.91
BS016	0.74	0.71	0.68	0.71	0.42	0.11	0.35	0.87	0.02	0.00	0.02	0.02	0.50	0.26	0.00	0.00	0.00	0.97
BS059	0.14	0.14	0.20	0.16	0.37	0.29	0.15	0.91	0.14	0.46	0.07	0.77	0.25	0.38	0.00	0.00	0.00	0.54
BS060	0.00	0.00	0.00	0.00	0.32	0.49	0.03	0.78	0.17	0.56	0.24	0.28	0.75	0.40	0.07	0.47	0.27	0.67
. . .	..	..	..	..	..	..	..	..	...	...	...	...	...	...	...	...	...	...
BS061	0.00	0.00	0.00	0.00	0.11	0.79	0.03	0.77	0.00	0.00	0.00	0.11	0.13	0.21	0.18	0.00	0.09	0.30
BS084	0.16	0.16	0.15	0.16	0.26	0.36	0.14	0.88	0.32	0.08	0.10	0.41	0.25	0.31	0.29	0.47	0.38	0.84
BS086	0.13	0.08	0.05	0.09	0.26	0.25	0.03	0.80	0.37	0.68	0.32	0.12	0.25	0.34	0.04	0.47	0.25	0.68
BS087	0.67	0.62	0.63	0.64	0.37	0.17	0.53	0.85	0.12	0.90	0.17	0.49	0.00	0.40	0.00	0.00	0.00	1.04
BS097	0.83	0.79	0.73	0.78	0.11	0.08	0.61	0.88	0.36	0.08	0.07	0.33	0.13	0.29	1.00	0.00	0.50	1.57

Table A.3. Value of the PVI2 metric for October 2023

	Outcome-vulnerability metrics							Outcome-vulnerability metrics (scaled)							
Station	MVM1	MVM2	DVM1	DVM2	FVM1	DTM2	DTM1	MVM1	MVM2	DVM1	DVM2	FVM1	DTM2	DTM1	PVI2
BS007	62	15	8	3	4	0	0	0.07	0.50	0.26	0.10	0.50	0.00	0.00	0.20
BS011	24	24	1	1	1	0	0	0.03	0.80	0.03	0.03	0.13	0.00	0.00	0.15
BS016	498	25	31	31	1	13	46	0.54	0.83	1.00	1.00	0.13	0.76	0.74	0.71
BS059	17	9	4	1	4	0	0	0.02	0.30	0.13	0.03	0.50	0.00	0.00	0.14
BS060	0	0	0	0	0	0	0	0.00	0.00	0.00	0.00	0.00	0.00	0.00	0.00
. . .	..	..	..	..	..	..	..	..	...	...	...	...	...	...	...
BS061	0	0	0	0	0	0	0	0.00	0.00	0.00	0.00	0.00	0.00	0.00	0.00
BS084	24	22	2	1	2	0	0	0.03	0.73	0.06	0.03	0.25	0.00	0.00	0.16
BS086	7	6	2	1	2	0	0	0.01	0.20	0.06	0.03	0.25	0.00	0.00	0.08
BS087	538	22	31	31	1	8	25	0.58	0.73	1.00	1.00	0.13	0.47	0.40	0.62
BS097	634	27	31	31	1	17	50	0.68	0.90	1.00	1.00	0.13	1.00	0.81	0.79

Table A.4. Value of the PVI3 metric for October 2023

	Outcome-vulnerability metrics							Outcome-vulnerability metrics (scaled)							
Station	MVM1	MVM2	DVM1	DVM2	FVM1	DTM2	DTM1	MVM1	MVM2	DVM1	DVM2	FVM1	DTM2	DTM1	PVI3
BS007	58	16	9	3	4	2	7	0.06	0.53	0.29	0.10	0.50	0.12	0.11	0.24
BS011	23	23	1	1	1	0	0	0.02	0.77	0.03	0.03	0.13	0.00	0.00	0.14
BS016	459	24	31	31	1	12	40	0.49	0.80	1.00	1.00	0.13	0.71	0.65	0.68
BS059	22	8	7	3	5	1	7	0.02	0.27	0.23	0.10	0.63	0.06	0.11	0.20
BS060	0	0	0	0	0	0	0	0.00	0.00	0.00	0.00	0.00	0.00	0.00	0.00
. . .	..	..	..	..	..	..	..	..	...	...	...	...	...	...	...
BS061	0	0	0	0	0	0	0	0.00	0.00	0.00	0.00	0.00	0.00	0.00	0.00
BS084	22	21	2	1	2	0	0	0.02	0.70	0.06	0.03	0.25	0.00	0.00	0.15
BS086	5	5	1	1	1	0	0	0.01	0.17	0.03	0.03	0.13	0.00	0.00	0.05
BS087	486	20	31	31	1	11	27	0.52	0.67	1.00	1.00	0.13	0.65	0.44	0.63
BS097	624	25	31	31	1	15	36	0.67	0.83	1.00	1.00	0.13	0.88	0.58	0.73

Table B.1. Value of the PVI1 metric for November 2023

	Outcome-vulnerability metrics							Outcome-vulnerability metrics (scaled)							
Station	MVM1	MVM2	DVM1	DVM2	FVM1	DTM2	DTM1	MVM1	MVM2	DVM1	DVM2	FVM1	DTM2	DTM1	PVI1
BS007	30	28	2	1	2	2	0	0.03	0.93	0.06	0.03	0.25	0.12	0.00	0.20
BS011	4	4	1	1	1	0	0	0.00	0.13	0.03	0.03	0.13	0.00	0.00	0.05
BS016	539	28	30	30	1	12	54	0.58	0.93	0.97	0.97	0.13	0.71	0.87	0.74
BS059	6	5	2	1	2	0	0	0.01	0.17	0.06	0.03	0.25	0.00	0.00	0.07
BS060	0	0	0	0	0	0	0	0.00	0.00	0.00	0.00	0.00	0.00	0.00	0.00
. . .	..	..	..	..	..	..	..	..	...	...	...	...	...	...	...
BS061	0	0	0	0	0	0	0	0.00	0.00	0.00	0.00	0.00	0.00	0.00	0.00
BS084	214	24	21	17	3	0	47	0.23	0.80	0.68	0.55	0.38	0.00	0.76	0.48
BS086	2	1	2	1	2	1	0	0.00	0.03	0.06	0.03	0.25	0.06	0.00	0.06
BS087	642	29	30	30	1	13	27	0.69	0.97	0.97	0.97	0.13	0.76	0.44	0.70
BS097	759	30	30	30	1	16	37	0.82	1.00	0.97	0.97	0.13	0.94	0.60	0.77

Table B.2. Value of PVI, VDF, VEF, and VI for November 2023

	Performances			OVI	Drivers									VDF	Exposures		VEF	VI
Station	PVI1	PVI2	PVI3	.	VDF1	VDF2	VDF3	VDF4	VDF5	VDF6	VDF7	VDF8	VDF9	.	VEF1	VEF2	.	.
BS007	0.20	0.17	0.18	0.19	0.26	0.46	0.08	0.80	0.36	0.44	0.12	0.29	0.63	0.38	0.04	0.00	0.02	0.59
BS011	0.05	0.05	0.04	0.05	0.32	0.63	0.05	0.84	0.21	0.58	1.00	0.00	0.63	0.47	0.00	0.47	0.23	0.75
BS016	0.74	0.71	0.68	0.71	0.32	0.13	0.35	0.83	0.00	0.00	-0.02	0.02	0.13	0.19	0.00	0.00	0.00	0.90
BS059	0.07	0.07	0.12	0.09	0.32	0.32	0.14	0.84	0.29	0.06	0.02	0.77	0.38	0.35	0.00	0.00	0.00	0.44
BS060	0.00	0.00	0.00	0.00	0.26	0.59	0.03	0.80	0.13	0.08	0.12	0.28	0.50	0.31	0.00	0.47	0.23	0.54
. . .	..	..	..	..	..	..	..	..	...	...	...	...	...	...	...	...	...	...
BS061	0.00	0.00	0.00	0.00	0.16	0.70	0.05	0.77	0.00	0.00	-0.02	0.11	0.38	0.24	0.14	0.00	0.07	0.31
BS084	0.48	0.43	0.38	0.43	0.26	0.13	0.25	0.94	0.00	0.00	0.00	0.41	0.13	0.24	0.00	0.47	0.23	0.90
BS086	0.06	0.09	0.08	0.08	0.32	0.41	0.03	0.78	0.39	0.92	0.32	0.12	0.13	0.38	0.07	0.47	0.27	0.72
BS087	0.70	0.64	0.63	0.66	0.21	0.07	0.51	0.86	0.10	0.48	0.05	0.49	0.13	0.32	0.00	0.00	0.00	0.98
BS097	0.77	0.74	0.70	0.74	0.05	0.07	0.99	0.70	0.36	0.20	0.15	0.33	0.25	0.34	0.14	0.00	0.07	1.15

Table B.3. Value of the PVI2 metric for November 2023

	Outcome-vulnerability metrics							Outcome-vulnerability metrics (scaled)							
Station	MVM1	MVM2	DVM1	DVM2	FVM1	DTM2	DTM1	MVM1	MVM2	DVM1	DVM2	FVM1	DTM2	DTM1	PVI2
BS007	26	25	2	1	2	0	0	0.03	0.83	0.06	0.03	0.25	0.00	0.00	0.17
BS011	5	5	1	1	1	0	0	0.01	0.17	0.03	0.03	0.13	0.00	0.00	0.05
BS016	470	25	30	30	1	13	52	0.51	0.83	0.97	0.97	0.13	0.76	0.84	0.71
BS059	9	5	2	1	2	0	0	0.01	0.17	0.06	0.03	0.25	0.00	0.00	0.07
BS060	0	0	0	0	0	0	0	0.00	0.00	0.00	0.00	0.00	0.00	0.00	0.00
. . .	..	..	..	..	..	..	..	..	...	...	...	...	...	...	...
BS061	0	0	0	0	0	0	0	0.00	0.00	0.00	0.00	0.00	0.00	0.00	0.00
BS084	172	21	19	17	3	0	36	0.19	0.70	0.61	0.55	0.38	0.00	0.58	0.43
BS086	8	5	3	2	2	0	1	0.01	0.17	0.10	0.06	0.25	0.00	0.02	0.09
BS087	569	26	30	30	1	8	27	0.61	0.87	0.97	0.97	0.13	0.47	0.44	0.64
BS097	681	27	30	30	1	17	32	0.73	0.90	0.97	0.97	0.13	1.00	0.52	0.74

Table B.4. Value of the PVI3 metric for November 2023

	Outcome-vulnerability metrics							Outcome-vulnerability metrics (scaled)							
Station	MVM1	MVM2	DVM1	DVM2	FVM1	DTM2	DTM1	MVM1	MVM2	DVM1	DVM2	FVM1	DTM2	DTM1	PVI3
BS007	26	24	2	1	2	2	0	0.03	0.80	0.06	0.03	0.25	0.12	0.00	0.18
BS011	3	3	1	1	1	0	0	0.00	0.10	0.03	0.03	0.13	0.00	0.00	0.04
BS016	435	23	30	30	1	12	46	0.47	0.77	0.97	0.97	0.13	0.71	0.74	0.68
BS059	13	6	4	2	3	1	0	0.01	0.20	0.13	0.06	0.38	0.06	0.00	0.12
BS060	0	0	0	0	0	0	0	0.00	0.00	0.00	0.00	0.00	0.00	0.00	0.00
. . .	..	..	..	..	..	..	..	..	...	...	...	...	...	...	...
BS061	0	0	0	0	0	0	0	0.00	0.00	0.00	0.00	0.00	0.00	0.00	0.00
BS084	152	19	17	10	4	0	30	0.16	0.63	0.55	0.32	0.50	0.00	0.48	0.38
BS086	10	4	3	2	2	0	0	0.01	0.13	0.10	0.06	0.25	0.00	0.00	0.08
BS087	518	24	30	30	1	11	23	0.56	0.80	0.97	0.97	0.13	0.65	0.37	0.63
BS097	652	25	30	30	1	15	24	0.70	0.83	0.97	0.97	0.13	0.88	0.39	0.70

Table C.1. Value of the PVI1 metric for December 2023

	Outcome-vulnerability metrics							Outcome-vulnerability metrics (scaled)							
Station	MVM1	MVM2	DVM1	DVM2	FVM1	DTM2	DTM1	MVM1	MVM2	DVM1	DVM2	FVM1	DTM2	DTM1	PVI1
BS007	90	30	3	3	1	2	0	0.10	1.00	0.10	0.10	0.13	0.12	0.00	0.22
BS011	0	0	0	0	0	0	0	0.00	0.00	0.00	0.00	0.00	0.00	0.00	0.00
BS016	608	26	31	31	1	12	46	0.65	0.87	1.00	1.00	0.13	0.71	0.74	0.73
BS059	41	16	8	3	5	0	16	0.04	0.53	0.26	0.10	0.63	0.00	0.26	0.26
BS060	0	0	0	0	0	0	0	0.00	0.00	0.00	0.00	0.00	0.00	0.00	0.00
. . .	..	..	..	..	..	..	..	..	...	...	...	...	...	...	...
BS061	0	0	0	0	0	0	0	0.00	0.00	0.00	0.00	0.00	0.00	0.00	0.00
BS084	701	27	31	31	1	0	38	0.75	0.90	1.00	1.00	0.13	0.00	0.61	0.63
BS086	0	0	0	0	0	1	0	0.00	0.00	0.00	0.00	0.00	0.06	0.00	0.01
BS087	624	27	31	31	1	13	50	0.67	0.90	1.00	1.00	0.13	0.76	0.81	0.75
BS097	388	30	15	12	2	16	19	0.42	1.00	0.48	0.39	0.25	0.94	0.31	0.54

Table C.2. Value of PVI, VDF, VEF, and VI for December 2023

	Performances			OVI	Drivers									VDF	Exposures		VEF	VI
Station	PVI1	PVI2	PVI3	.	VDF1	VDF2	VDF3	VDF4	VDF5	VDF6	VDF7	VDF8	VDF9	.	VEF1	VEF2	.	.
BS007	0.22	0.19	0.19	0.20	0.26	0.47	0.08	0.81	0.33	0.38	0.22	0.29	0.75	0.40	0.29	0.00	0.14	0.74
BS011	0.00	0.00	0.00	0.00	0.21	0.62	0.06	0.87	0.20	0.68	0.44	0.00	0.13	0.36	0.04	0.47	0.25	0.61
BS016	0.73	0.71	0.67	0.70	0.29	0.13	0.43	0.91	0.00	0.00	0.00	0.02	0.13	0.21	0.00	0.00	0.00	0.92
BS059	0.26	0.22	0.25	0.24	0.32	0.30	0.15	0.86	0.00	0.00	-0.02	0.77	0.38	0.31	0.07	0.00	0.04	0.59
BS060	0.00	0.00	0.00	0.00	0.26	0.58	0.03	0.76	0.40	0.12	0.15	0.28	0.25	0.31	0.00	0.47	0.23	0.55
. . .	..	..	..	..	..	..	..	..	...	...	...	...	...	...	...	...	...	...
BS061	0.00	0.00	0.00	0.00	0.16	0.66	0.05	0.77	0.00	0.00	-0.02	0.11	0.50	0.25	0.04	0.00	0.02	0.26
BS084	0.63	0.59	0.58	0.60	0.26	0.07	0.51	0.94	0.00	0.00	0.00	0.41	0.13	0.26	0.04	0.47	0.25	1.11
BS086	0.01	0.00	0.00	0.00	0.21	0.56	0.01	0.82	0.27	0.28	0.20	0.12	0.25	0.30	0.00	0.47	0.23	0.54
BS087	0.75	0.66	0.66	0.69	0.16	0.09	0.47	0.83	0.15	0.10	0.02	0.49	0.13	0.27	0.00	0.00	0.00	0.96
BS097	0.54	0.51	0.48	0.51	0.21	0.23	0.11	0.78	0.70	0.00	0.05	0.33	0.25	0.30	0.11	0.00	0.05	0.86

Table C.3. Value of the PVI2 metric for December 2023

	Outcome-vulnerability metrics							Outcome-vulnerability metrics (scaled)							
Station	MVM1	MVM2	DVM1	DVM2	FVM1	DTM2	DTM1	MVM1	MVM2	DVM1	DVM2	FVM1	DTM2	DTM1	PVI2
BS007	81	27	3	3	1	0	0	0.09	0.90	0.10	0.10	0.13	0.00	0.00	0.19
BS011	0	0	0	0	0	0	0	0.00	0.00	0.00	0.00	0.00	0.00	0.00	0.00
BS016	525	23	31	31	1	13	45	0.57	0.77	1.00	1.00	0.13	0.76	0.73	0.71
BS059	39	13	8	4	5	0	3	0.04	0.43	0.26	0.13	0.63	0.00	0.05	0.22
BS060	0	0	0	0	0	0	0	0.00	0.00	0.00	0.00	0.00	0.00	0.00	0.00
. . .	..	..	..	..	..	..	..	..	...	...	...	...	...	...	...
BS061	0	0	0	0	0	0	0	0.00	0.00	0.00	0.00	0.00	0.00	0.00	0.00
BS084	612	24	31	31	1	0	36	0.66	0.80	1.00	1.00	0.13	0.00	0.58	0.59
BS086	0	0	0	0	0	0	0	0.00	0.00	0.00	0.00	0.00	0.00	0.00	0.00
BS087	554	24	31	31	1	8	38	0.60	0.80	1.00	1.00	0.13	0.47	0.61	0.66
BS097	351	27	15	12	2	17	11	0.38	0.90	0.48	0.39	0.25	1.00	0.18	0.51

Table C.4. Value of the PVI3 metric for December 2023

	Outcome-vulnerability metrics							Outcome-vulnerability metrics (scaled)							
Station	MVM1	MVM2	DVM1	DVM2	FVM1	DTM2	DTM1	MVM1	MVM2	DVM1	DVM2	FVM1	DTM2	DTM1	PVI3
BS007	75	25	3	3	1	2	0	0.08	0.83	0.10	0.10	0.13	0.12	0.00	0.19
BS011	0	0	0	0	0	0	0	0.00	0.00	0.00	0.00	0.00	0.00	0.00	0.00
BS016	489	21	31	31	1	12	41	0.53	0.70	1.00	1.00	0.13	0.71	0.66	0.67
BS059	46	12	10	6	5	1	8	0.05	0.40	0.32	0.19	0.63	0.06	0.13	0.25
BS060	0	0	0	0	0	0	0	0.00	0.00	0.00	0.00	0.00	0.00	0.00	0.00
. . .	..	..	..	..	..	..	..	..	...	...	...	...	...	...	...
BS061	0	0	0	0	0	0	0	0.00	0.00	0.00	0.00	0.00	0.00	0.00	0.00
BS084	550	22	31	31	1	0	38	0.59	0.73	1.00	1.00	0.13	0.00	0.61	0.58
BS086	0	0	0	0	0	0	0	0.00	0.00	0.00	0.00	0.00	0.00	0.00	0.00
BS087	506	22	31	31	1	11	34	0.54	0.73	1.00	1.00	0.13	0.65	0.55	0.66
BS097	337	25	15	12	2	15	10	0.36	0.83	0.48	0.39	0.25	0.88	0.16	0.48

Table D.1. Vulnerability score of 34 locations in October, November, and December 2023

No	Station	October	November	December	No	Station	October	November	December
1	BS007	0.575	0.587	0.741	18	BS151	0.673	0.504	0.738
2	BS011	0.912	0.753	0.606	19	BS162	0.717	0.687	0.728
3	BS016	0.971	0.903	0.915	20	BS199	0.669	0.684	0.620
4	BS059	0.538	0.439	0.585	21	BS218	0.723	0.677	0.651
5	BS060	0.670	0.543	0.547	22	BS221	1.047	1.330	1.596
6	BS061	0.303	0.308	0.264	23	BS229	0.535	0.557	0.590
7	BS084	0.843	0.899	1.109	24	BS233	0.730	0.459	0.401
8	BS086	0.680	0.723	0.537	25	BS247	0.384	0.427	0.599
9	BS087	1.038	0.977	0.960	26	BS253	0.258	0.488	0.448
10	BS097	1.574	1.153	0.860	27	BS259	0.848	0.881	0.801
11	BS102	0.280	0.221	0.231	28	BS264	0.612	0.791	0.993
12	BS105	0.774	0.773	0.825	29	BS268	0.985	0.957	0.564
13	BS121	0.904	0.666	0.592	30	BS270	0.860	0.687	1.186
14	BS126	0.486	0.532	0.412	31	BS279	1.004	1.042	1.093
15	BS128	0.738	1.224	0.803	32	BS284	1.319	1.191	1.269
16	BS137	0.779	0.957	0.810	33	BS289	1.416	1.444	1.333
17	BS142	0.572	0.835	0.853	34	BS290	1.305	1.255	1.174
